# Association of leuko-glycemic index with mortality in critically ill stroke patients: analysis from the MIMIC-IV database and an institutional cohort

**DOI:** 10.3389/fmed.2026.1755389

**Published:** 2026-02-11

**Authors:** Yu Wang, Wentao Wang, Xiaochuan Sun, Xiaomin Yang, Jun Chen, Dan Xu

**Affiliations:** 1 Department of Neurosurgery, The First Affiliated Hospital of Chongqing Medical University, Yuzhong, Chongqing, China; 2 International Medical College, Chongqing Medical University, Yuzhong, Chongqing, China

**Keywords:** all-cause mortality, critical illness, leuko-glycemic index, MIMIC-IV database, prognosis, stroke

## Abstract

**Background:**

The leuko-glycemic index (LGI), combining white blood cell count and blood glucose, reflects systemic inflammation and stress hyperglycemia. Its prognostic value in critically ill stroke patients remains unclear. This study evaluated the association between LGI and mortality using the MIMIC-IV database and an independent institutional cohort.

**Methods:**

Patients with acute stroke were identified from MIMIC-IV (version 3.1) and a neurosurgical intensive care unit cohort. LGI was calculated as white blood cell count (×10^9^/L) × blood glucose (mg/dL)/1,000 using measurements within the first 24 h after ICU admission and categorized into quartiles. The primary outcome was 28-day mortality. Secondary outcomes included in-hospital mortality in both cohorts and 365-day mortality in MIMIC-IV. Multivariable Cox models were used to assess the association between LGI and mortality; in MIMIC-IV, spline-based analyses further explored the dose–response pattern, and subgroup analyses were performed.

**Results:**

A total of 5,267 patients from MIMIC-IV and 424 from the institutional cohort were included. In MIMIC-IV, after multivariable adjustment, patients in the highest LGI quartile had more than threefold higher risk of 28-day mortality compared with the lowest quartile (hazard ratio [HR] 3.259, 95% confidence interval [CI] 2.568–4.138; *p* for trend < 0.001) and higher 365-day mortality (HR 2.703, 95% CI 2.203–3.317; *p* for trend < 0.001). LGI was also positively associated with 28-day mortality in the institutional cohort (adjusted HR 1.351, 95% CI 1.090–1.675), and in-hospital mortality rates were likewise highest in the top LGI quartile. Exploratory spline analyses in MIMIC-IV showed that risk increased steeply from low to moderate LGI and then rose more gradually at higher levels. Subgroup analyses suggested broadly consistent positive associations across most clinical strata.

**Conclusion:**

LGI was significantly associated with mortality in critically ill stroke patients across two independent cohorts and may serve as a simple, readily available biomarker to aid early risk stratification.

## 1 Introduction

Stroke is a major type of cerebrovascular disease and, together with other cardiovascular conditions, remains a leading cause of death and disability in adults (1). With global population aging, the burden of both ischemic and hemorrhagic stroke is increasing, and critically ill stroke patients impose a substantial demand on intensive care resources. Therefore, simple, low-cost, and easily applicable prognostic markers are urgently needed to identify high-risk patients and guide early risk stratification in clinical practice (2–4). Increasing evidence suggests that both metabolic disturbances and inflammatory responses play pivotal roles in the progression of acute stroke and its associated adverse outcomes. Hyperglycemia, leukocytosis, and systemic inflammatory activation are common physiological stress responses in critically ill patients and have each been reported to be associated with poor neurological recovery and increased mortality (5–7).

The leuko-glycemic index (LGI), calculated as the product of white blood cell (WBC) and blood glucose level, has emerged as a composite marker reflecting the interaction between inflammation and metabolic dysregulation. Prior studies have demonstrated that LGI is closely associated with unfavorable outcomes in several acute conditions, including severe infections, trauma, and cardiovascular disease (8–10). Elevated glucose levels can enhance leukocyte adhesion, chemotaxis, and oxidative activity, thereby amplifying the inflammatory cascade and exacerbating organ injury (11, 12). Given that stroke triggers a complex neuroinflammatory response and is frequently accompanied by stress-induced hyperglycemia (13–15), LGI may serve as a more comprehensive indicator of disease severity than either parameter alone.

Although LGI has shown prognostic value in other critical illnesses, its relationship with clinical outcomes among critically ill stroke patients remains uncertain. Existing research primarily focuses on either hyperglycemia or leukocytosis independently, while studies evaluating their combined effect through LGI are scarce. Furthermore, critically ill stroke patients often present with substantial heterogeneity in metabolic and inflammatory status, highlighting the need for reliable, easily obtainable prognostic biomarkers to support early risk stratification and clinical decision-making (16–18).

In light of these gaps, the present study aimed to investigate the association between LGI and mortality in critically ill stroke patients using data from both the Medical Information Mart for Intensive Care IV (MIMIC-IV) database and a Chinese institutional cohort. By evaluating the association between LGI and short-term (28-day) and long-term (365-day) all-cause mortality, this study sought to determine whether LGI could serve as a practical, cost-effective prognostic indicator to identify high-risk patients who may benefit from intensified monitoring and targeted interventions.

## 2 Methods and materials

### 2.1 Study population

This retrospective cohort study investigated critically ill stroke patients from two independent sources: the MIMIC-IV database and a Chinese institutional intensive care unit (ICU) cohort. Data from MIMIC-IV (version 3.1) covered ICU admissions between 2008 and 2019, whereas the institutional cohort included patients admitted between October 2023 and September 2025.

In the MIMIC-IV cohort, ICU patients with a primary diagnosis of acute stroke were identified using International Classification of Diseases, 9th and 10th Revision (ICD-9/10) codes. Eligible patients were required to be ≥18 years old at ICU admission, to have the first ICU admission during the index hospitalization, and to have documented WBC and blood glucose values within the first 24 h after ICU admission to allow calculation of LGI. Patients with pregnancy, active malignant disease, or missing WBC or blood glucose measurements within the first 24 h after ICU admission were excluded. After applying these criteria, 5,267 adult critically ill stroke patients were included in the final MIMIC-IV cohort. Access to the MIMIC-IV database is granted to individuals who have completed the Collaborative Institutional Training Initiative (CITI) program. One author (Yu Wang; certification number: 70805438) completed the required course and was granted access, which was used for data extraction in this study. The MIMIC-IV database contains de-identified electronic health records from the intensive care units of Beth Israel Deaconess Medical Center (19), and all personal identifiers have been removed and replaced with randomly generated codes; therefore, additional institutional review board approval and individual informed consent were not required.

For the institutional cohort, we retrospectively screened consecutive patients with acute stroke who were admitted to the neurosurgical intensive care unit (NICU) of the First Affiliated Hospital of Chongqing Medical University during the study period. The definition of stroke in the institutional cohort was based on clinicians’ comprehensive assessment of imaging findings and clinical features. Cases were then identified in the electronic medical record system using diagnostic codes and discharge diagnoses consistent with acute stroke. The same eligibility criteria as in the MIMIC-IV cohort were applied: age ≥18 years at NICU admission; first NICU admission during the study period; and availability of WBC and blood glucose measurements within the first 24 h after NICU admission. Patients with pregnancy, active malignant disease, or missing WBC or blood glucose measurements within the first 24 h after NICU admission were excluded. After screening, 424 critically ill stroke patients formed the institutional cohort. This study was approved by the local ethics committee, and the requirement for written informed consent was waived because of its retrospective design and use of anonymized data.

### 2.2 Data extraction

For the MIMIC-IV cohort, data were extracted using PostgreSQL (version 13.7.2) and Navicat Premium (version 15) by running Structured Query Language (SQL) queries. Clinical variables within the first 24 h after ICU admission were collected and included: demographic data (age, sex, and height); comorbidities (hypertension, diabetes mellitus, myocardial infarction, heart failure, renal disease, liver disease, and sepsis); vital signs (heart rate, respiratory rate, systolic, diastolic and mean blood pressure, and body temperature); laboratory parameters (WBC, hemoglobin, platelet count, blood urea nitrogen, serum creatinine, serum sodium, potassium, chloride, calcium, anion gap, bicarbonate, glucose, international normalized ratio [INR], and activated partial thromboplastin time [aPTT]); and ICU severity and treatments, including the Glasgow Coma Scale (GCS) score (20) and the use of vasopressors, insulin therapy, statins, and mechanical ventilation. For the institutional cohort, baseline clinical data were retrospectively retrieved from the hospital electronic medical record system using the same time window (within 24 h of NICU admission) and, as far as possible, the same variable definitions as in the MIMIC-IV cohort. Demographics, comorbidities, vital signs, laboratory measurements, and treatments were collected in a standardized format to ensure comparability between the two cohorts.

The LGI was calculated using the formula “LGI = WBC (×10^9^/L) × blood glucose (mg/dL)/1000” (8, 9). When multiple WBC or glucose measurements were available within the first 24 h after ICU/NICU admission, LGI was calculated using the first available (i.e., first recorded) WBC and the first available blood glucose within that 24-h window; the two values were not required to be from the same blood draw or recorded at the identical time. In both cohorts, patients were categorized into LGI quartiles (Q1–Q4) at admission, with Q1 serving as the reference group for descriptive analyses and Cox models. To address missing data in the MIMIC-IV cohort, variables with more than 20% missingness (height, missing in 54.2% of patients) were excluded from multivariable modeling, whereas variables with 20% or less missingness were handled using multiple imputation. Details of the imputation procedure (variables included, handling of categorical variables, and key settings) are provided in the Statistical analysis section (2.4).

### 2.3 Clinical outcomes

The primary outcome of this study was all-cause mortality within 28 days after ICU admission. The secondary outcomes included all-cause mortality within 365 days and in-hospital mortality. Mortality information in MIMIC-IV was derived from hospital records and validated date-of-death fields provided in the database documentation. For the institutional cohort, all-cause mortality data were extracted from the hospital electronic medical record system and supplemented by follow-up records. Because long-term (365-day) follow-up data were not available for the institutional cohort, only the primary outcome (28-day mortality) was analyzed in this cohort.

### 2.4 Statistical analysis

Continuous variables were summarized as medians with interquartile ranges (IQR), while categorical variables were presented as counts and percentages. The Kolmogorov–Smirnov test was applied to assess the normality of continuous variables. Group comparisons were performed using the Kruskal–Wallis test for continuous variables and Pearson’s chi-square test or Fisher’s exact test for categorical variables, as appropriate. Baseline characteristics across LGI quartiles were compared separately in the MIMIC-IV and institutional cohorts.

Kaplan–Meier survival curves were constructed to compare the incidence of 28-day and 365-day mortality across LGI quartiles in the MIMIC-IV cohort, and differences among groups were assessed using log-rank tests. For the institutional cohort, Kaplan–Meier curves for 28-day mortality across LGI quartiles were also generated. Cox proportional hazards regression models were used in both cohorts to estimate hazard ratios (HRs) and 95% confidence intervals (CIs) for the association between LGI and mortality outcomes. In these models, LGI was analyzed both as a continuous variable and as a categorical variable based on quartiles, with Q1 serving as the reference group. Potential confounders were identified based on univariable analyses (*p* < 0.05) and clinical relevance. Three Cox proportional hazards models were constructed: Model 1 was unadjusted; Model 2 was adjusted for age and sex; Model 3 was further adjusted for GCS score and mechanical ventilation. The extent of missing data for each study variable in the MIMIC-IV cohort is summarized in Supplementary Table 1. In the institutional cohort, there were no missing values for the variables included in the analyses; therefore, no imputation was performed and all analyses were conducted using complete data in the institutional cohort. As described above, in the MIMIC-IV cohort, variables with more than 20% missingness (e.g., height) were not included in the multivariable models. For variables with 20% or less missingness in the MIMIC-IV cohort, multiple imputation by chained equations (MICE) was performed using the mice package (version 3.15.0) in R (21). The imputation model included all variables listed in Table 1 (demographics, vital signs, laboratory tests, comorbidities, and treatments) as well as the outcome variables (28-day and 365-day mortality status and time-to-event) to make the Missing at Random (MAR) assumption more plausible. Predictive Mean Matching (PMM) was used for continuous variables and logistic regression for binary categorical variables. Five imputed datasets were generated, with the imputation process running for 10 iterations per dataset to achieve convergence. The results from analyses conducted on each imputed dataset were pooled using Rubin’s rules to obtain final parameter estimates and standard errors. As a sensitivity analysis, we additionally performed a complete-case analysis in the MIMIC-IV cohort, restricting Models 1–3 to the same complete-case subset defined by complete data on all covariates included in Model 3; results are reported in Supplementary Table 2. The proportional hazards assumption was assessed using Schoenfeld residual plots and Schoenfeld residual-based tests. For the LGI term in the primary 28-day mortality Cox model (Model 3) with LGI entered as a continuous term, the Schoenfeld residual-based test was not significant in either cohort (MIMIC-IV: *p* = 0.111; institutional cohort: *p* = 0.794; Supplementary Figure 2).

**Table 1:** Characteristics and outcomes of participants categorized by LGI in the MIMIC-IV cohort.

Categories	Overall (*N* = 5,267)	Q1 (*N* = 1,316)	Q2 (*N* = 1,316)	Q3 (*N* = 1,316)	Q4 (*N* = 1,319)	*p* value
LGI	1.36 (0.91–2.07)	0.69 (0.55–0.80)	1.13 (1.02–1.24)	1.65 (1.50–1.84)	2.77 (2.35–3.58)	< 0.001
Demographic						
Age (years)	73.00 (62.00–82.00)	73.00 (62.00–83.00)	73.00 (62.00–83.00)	72.00 (62.00–82.00)	72.00 (63.00–81.00)	0.677
Male, *n* (%)	2,782.00 (52.82%)	653.00 (49.62%)	707.00 (53.72%)	732.00 (55.62%)	690.00 (52.31%)	0.018
Clinical severity						
GCS	15.00 (13.00–15.00)	15.00 (13.00–15.00)	15.00 (13.00–15.00)	14.00 (13.00–15.00)	15.00 (12.00–15.00)	0.014
Vital signs						
Heart rate, beats/min	79.68 (70.92–90.08)	76.17 (67.42–86.83)	77.45 (69.70–86.80)	81.23 (73.34–91.62)	84.29 (75.39–95.00)	< 0.001
SBP, mmHg	121.38 (108.50–135.17)	122.50 (109.72–135.10)	122.74 (109.28–136.74)	121.16 (108.42–135.02)	119.00 (107.29–134.21)	< 0.001
DBP, mmHg	64.96 (57.36–74.12)	66.50 (58.59–75.55)	65.49 (57.67–74.91)	64.58 (57.29–73.59)	63.28 (56.26–72.00)	< 0.001
MBP, mmHg	79.10 (71.25–88.38)	80.81 (72.36–89.67)	79.90 (71.04–89.36)	78.69 (71.13–87.66)	77.60 (70.20–86.85)	< 0.001
RR, breaths/min	18.36 (16.41–20.71)	17.91 (16.15–19.88)	17.96 (16.25–19.96)	18.48 (16.49–20.84)	19.30 (17.00–22.08)	< 0.001
Temperature, °C	36.81 (36.61–37.07)	36.76 (36.60–36.97)	36.79 (36.60–37.03)	36.83 (36.61–37.11)	36.86 (36.62–37.17)	< 0.001
Laboratory tests						
Hemoglobin, g/dL	10.96 (9.45–12.50)	10.85 (9.30–12.50)	10.99 (9.50–12.55)	11.05 (9.60–12.50)	10.96 (9.28–12.53)	0.138
Platelet count, 10^9^/L	193.50 (148.00–250.67)	174.50 (131.75–221.00)	193.63 (147.59–245.00)	199.00 (154.00–256.34)	214.00 (159.67–274.00)	< 0.001
WBC, 10^9^/L	10.45 (7.70–13.90)	6.50 (5.35–7.60)	9.36 (8.10–10.80)	12.10 (10.40–14.00)	16.20 (13.30–20.00)	< 0.001
Anion gap, mmol/L	14.00 (12.00–16.00)	13.00 (11.00–15.00)	13.50 (11.84–15.50)	14.00 (12.00–16.00)	15.00 (13.00–17.33)	< 0.001
Bicarbonate, mmol/L	23.00 (21.00–25.50)	24.00 (22.00–26.00)	23.88 (21.67–26.00)	23.00 (21.00–25.33)	22.00 (19.50–24.50)	< 0.001
BUN, mmol/L	6.78 (4.82–11.07)	6.25 (4.49–10.00)	6.43 (4.64–10.00)	6.60 (5.00–10.53)	8.21 (5.53–13.45)	< 0.001
Serum creatinine, μmol/L	88.4 (66.3–129.9)	79.6 (61.9–118.5)	84.0 (66.3–120.2)	88.4 (66.3–128.2)	97.2 (70.7–152.0)	< 0.001
Calcium, mmol/L	2.12 (2.01–2.23)	2.15 (2.02–2.25)	2.15 (2.03–2.25)	2.11 (2.00–2.23)	2.11 (2.00–2.22)	< 0.001
Chloride, mmol/L	104.67 (101.00–108.00)	105.00 (102.00–108.00)	104.90 (101.50–107.50)	105.00 (101.00–108.00)	104.33 (100.50–108.00)	0.097
Glucose, mg/dL	128.00 (106.00–159.33)	102.00 (91.50–115.63)	119.00 (105.00–137.00)	136.00 (118.50–158.50)	179.50 (144.50–228.00)	< 0.001
Sodium, mmol/L	139.00 (137.00–141.50)	140.00 (137.00–142.00)	139.00 (137.00–141.00)	139.00 (136.67–141.00)	139.00 (136.00–141.50)	< 0.001
Potassium, mmol/L	4.10 (3.77–4.45)	4.00 (3.70–4.35)	4.05 (3.75–4.40)	4.10 (3.80–4.50)	4.15 (3.80–4.57)	< 0.001
INR	1.20 (1.10–1.43)	1.20 (1.10–1.40)	1.20 (1.10–1.40)	1.23 (1.10–1.40)	1.25 (1.10–1.50)	< 0.001
aPTT, s	30.70 (27.10–38.13)	31.30 (27.90–38.90)	30.40 (27.13–36.20)	30.30 (26.88–37.32)	30.80 (26.65–40.10)	< 0.001
Comorbidities						
MI, *n* (%)	389.00 (7.39%)	53.00 (4.03%)	81.00 (6.16%)	105.00 (7.98%)	150.00 (11.37%)	< 0.001
HF, *n* (%)	1,620.00 (30.76%)	371.00 (28.19%)	375.00 (28.50%)	419.00 (31.84%)	455.00 (34.50%)	< 0.001
Hypertension, *n* (%)	2,696.00 (51.19%)	641.00 (48.71%)	710.00 (53.95%)	699.00 (53.12%)	646.00 (48.98%)	0.008
LD, *n* (%)	233.00 (4.42%)	87.00 (6.61%)	41.00 (3.12%)	55.00 (4.18%)	50.00 (3.79%)	< 0.001
DM, *n* (%)	1,905.00 (36.17%)	471.00 (35.79%)	449.00 (34.12%)	486.00 (36.93%)	499.00 (37.83%)	0.223
RD, *n* (%)	2,010.00 (38.16%)	429.00 (32.60%)	439.00 (33.36%)	509.00 (38.68%)	633.00 (47.99%)	< 0.001
Sepsis, *n* (%)	588.00 (11.16%)	83.00 (6.31%)	114.00 (8.66%)	138.00 (10.49%)	253.00 (19.18%)	< 0.001
Treatment						
Statin, *n* (%)	1,018.00 (19.33%)	251.00 (19.07%)	267.00 (20.29%)	269.00 (20.44%)	231.00 (17.51%)	0.198
Vasopressor, *n* (%)	1,995.00 (37.88%)	315.00 (23.94%)	486.00 (36.93%)	554.00 (42.10%)	640.00 (48.52%)	< 0.001
Insulin, *n* (%)	218.00 (4.14%)	23.00 (1.75%)	27.00 (2.05%)	50.00 (3.80%)	118.00 (8.95%)	< 0.001
MV, *n* (%)	3,276.00 (62.20%)	657.00 (49.92%)	791.00 (60.11%)	905.00 (68.77%)	923.00 (69.98%)	< 0.001
Outcomes						
28-day mortality, *n* (%)	707.00 (13.42%)	88.00 (6.69%)	121.00 (9.19%)	187.00 (14.21%)	311.00 (23.58%)	< 0.001
365-day mortality, *n* (%)	850.00 (16.14%)	128.00 (9.73%)	156.00 (11.85%)	217.00 (16.49%)	349.00 (26.46%)	< 0.001
Hospital mortality, *n* (%)	312.00 (5.92%)	63.00 (4.79%)	67.00 (5.09%)	81.00 (6.16%)	101.00 (7.66%)	0.008
LOS hospital, days	7.12 (3.98–12.67)	5.93 (3.14–10.88)	7.05 (4.03–12.43)	7.64 (4.59–12.92)	7.90 (4.47–13.98)	< 0.001

LGI, leuko-glycemic index; GCS, Glasgow Coma Scale; SBP, systolic blood pressure; DBP, diastolic blood pressure; MBP, mean blood pressure; RR, respiratory rate; WBC, white blood cell; BUN, blood urea nitrogen; INR, international normalized ratio; aPTT, activated partial thromboplastin time; LOS, length of stay; MI, myocardial infarction; HF, heart failure; LD, liver disease; DM, diabetes mellitus; RD, renal disease; MV, mechanical ventilation.

In the MIMIC-IV cohort, additional analyses were undertaken to describe the shape of the association between LGI and mortality. Restricted cubic spline (RCS) functions with four knots (at the 5th, 35th, 65th, and 95th percentiles of the LGI distribution) were fitted in Cox models for 28-day and 365-day mortality to visualize the dose–response pattern without imposing a specific functional form. In addition, a piecewise Cox proportional hazards model with a single data-driven change point (K) was fitted to provide a parsimonious summary of the association in lower and higher LGI ranges; the estimated change point was treated as a statistical approximation that aids interpretation rather than as a precise biological threshold. Linear and piecewise models were compared using log-likelihood ratio tests. Nonlinear and threshold-effect analyses were not applied to the institutional cohort because of its smaller sample size.

Trend tests across LGI quartiles were performed by entering the quartile number as a continuous variable in the regression model. Prespecified subgroup analyses were conducted in the MIMIC-IV cohort to evaluate the robustness of the association between LGI and mortality across strata of age (<65 vs. ≥65 years), sex, GCS score (≤8 vs. >8), history of myocardial infarction, diabetes mellitus, vasopressor use, insulin therapy, and mechanical ventilation. Interaction terms between LGI and each subgroup variable were examined using likelihood ratio tests to investigate whether the association differed across subgroups. To further assess robustness within the main exposure range, key subgroup analyses were repeated in sensitivity analyses restricted to patients with LGI values below the estimated change points identified in the piecewise Cox models. Interaction analyses were considered exploratory, and no formal adjustment for multiple comparisons was performed. All analyses were conducted using R software (version 4.2.3) and SPSS 22.0 (IBM Corp., Armonk, NY, USA). Two-sided *p* values < 0.05 were considered statistically significant.

## 3 Results

### 3.1 Comparison of patients’ baseline information

**Figure 1:**
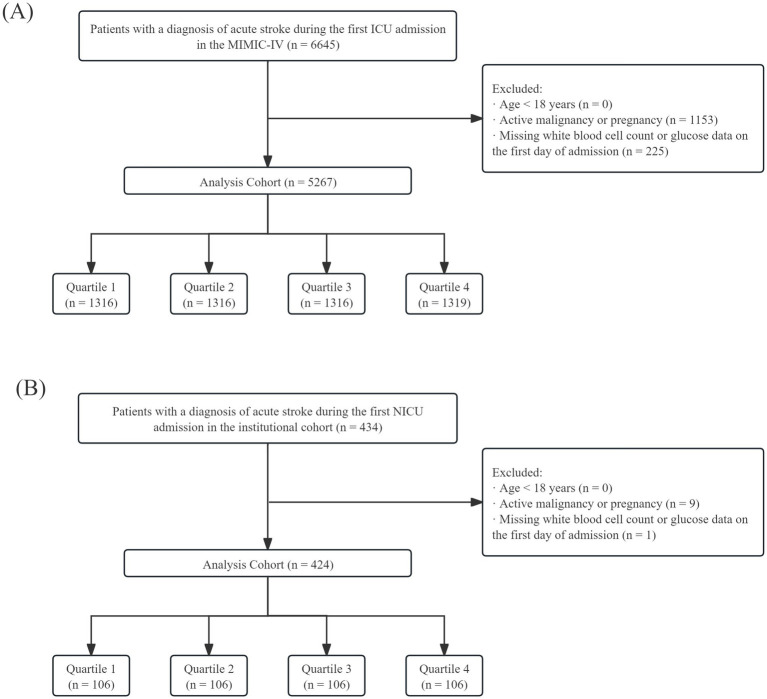
Flowchart of patient selection in the MIMIC-IV cohort **(A)** and in the institutional cohort **(B)**. ICU, intensive care unit; NICU, neurosurgical intensive care unit.

A total of 5,267 adult critically ill stroke patients from the MIMIC-IV cohort and 424 patients from the institutional cohort at a Chinese neurosurgical ICU were included in the final analysis (Figure 1). In both cohorts, baseline characteristics were compared across LGI quartiles (Q1–Q4), as summarized in Table 1 (MIMIC-IV cohort) and Table 2 (institutional cohort). Details of missingness for variables in the MIMIC-IV cohort are provided in Supplementary Table 1. In the institutional cohort, no missing data were observed for the variables analyzed.

**Table 2:** Characteristics and outcomes of participants categorized by LGI in the institutional cohort.

Categories	Overall (*N* = 424)	Q1 (*N* = 106)	Q2 (*N* = 106)	Q3 (*N* = 106)	Q4 (*N* = 106)	*p* value
LGI	1.13 (0.80–1.72)	0.60 (0.50–0.70)	0.97 (0.90–1.04)	1.42 (1.28–1.53)	2.23 (1.95–2.99)	< 0.001
Demographic						
Age (years)	60.00 (52.00–69.00)	57.00 (50.00–68.00)	61.00 (53.00–72.00)	61.50 (53.00–70.00)	59.00 (52.00–69.00)	0.062
Male, *n* (%)	207.00 (48.82%)	55.00 (51.89%)	52.00 (49.06%)	51.00 (48.11%)	49.00 (46.23%)	0.871
Clinical severity						
GCS	13.00 (11.00–14.00)	13.00 (11.00–15.00)	13.00 (12.00–15.00)	13.00 (11.00–14.00)	12.00 (8.00–14.00)	< 0.001
Vital signs						
Heart rate, beats/min	79.00 (70.00–89.00)	77.00 (70.00–87.00)	78.00 (66.00–88.00)	78.00 (70.00–89.00)	82.50 (73.00–94.00)	0.050
SBP, mmHg	144.00 (131.00–159.00)	139.50 (127.00–156.00)	142.50 (127.00–157.00)	143.50 (133.00–159.00)	150.50 (135.00–166.00)	0.006
DBP, mmHg	86.00 (76.00–96.00)	87.00 (77.00–96.00)	83.00 (74.00–92.00)	85.50 (79.00–93.00)	87.00 (76.00–99.00)	0.194
MBP, mmHg	105.17 (95.33–116.00)	105.33 (93.33–112.33)	101.00 (93.00–113.33)	105.00 (97.33–116.67)	107.50 (97.67–118.00)	0.049
RR, breaths/min	18.00 (16.00–20.00)	19.00 (17.00–20.00)	18.00 (16.00–20.00)	17.00 (15.00–20.00)	18.00 (15.00–20.00)	0.185
Temperature, °C	36.50 (36.40–36.70)	36.50 (36.40–36.70)	36.50 (36.40–36.70)	36.50 (36.50–36.70)	36.55 (36.40–36.70)	0.518
Laboratory tests						
Hemoglobin, g/dL	13.25 (12.10–14.40)	13.20 (12.20–14.30)	13.15 (12.00–14.40)	13.35 (12.10–14.30)	13.50 (12.20–14.60)	0.776
Platelet count, 10^9^/L	202.00 (164.00–245.00)	188.00 (151.00–240.00)	182.50 (145.00–228.00)	208.50 (174.00–253.00)	221.50 (182.00–265.00)	< 0.001
WBC, 10^9^/L	8.80 (6.80–11.68)	6.02 (5.18–6.98)	8.32 (6.71–9.20)	10.35 (8.78–11.59)	14.20 (11.69–17.35)	< 0.001
Anion gap, mmol/L	15.30 (13.30–17.20)	14.05 (11.80–15.80)	15.00 (13.10–16.80)	15.30 (13.40–17.10)	17.10 (15.10–19.30)	< 0.001
Bicarbonate, mmol/L	23.00 (21.00–25.10)	23.90 (22.10–25.90)	22.90 (21.20–24.90)	22.65 (21.10–25.10)	21.90 (20.00–24.60)	< 0.001
BUN, mmol/L	5.10 (4.00–6.20)	5.10 (3.90–6.00)	4.80 (3.70–6.50)	5.40 (4.20–6.40)	4.90 (4.20–6.00)	0.237
Serum creatinine, μmol/L	61.00 (50.00–74.00)	64.00 (54.00–74.00)	57.00 (51.00–73.00)	61.00 (51.00–75.00)	60.50 (48.00–74.00)	0.274
Calcium, mmol/L	2.24 (2.16–2.31)	2.23 (2.17–2.30)	2.24 (2.16–2.31)	2.24 (2.14–2.31)	2.24 (2.17–2.32)	0.899
Chloride, mmol/L	105.00 (102.00–107.00)	105.00 (103.00–108.00)	105.00 (103.00–108.00)	104.00 (102.00–107.00)	103.00 (101.00–106.00)	< 0.001
Glucose, mg/dL	126.00 (104.40–160.20)	94.50 (86.40–106.20)	117.00 (108.00–133.20)	131.40 (126.00–154.80)	180.00 (145.80–219.60)	< 0.001
Sodium, mmol/L	139.50 (137.00–141.00)	140.00 (138.00–142.00)	140.00 (137.00–141.00)	139.00 (137.00–141.00)	139.00 (137.00–141.00)	0.339
Potassium, mmol/L	3.80 (3.50–4.00)	3.90 (3.60–4.10)	3.90 (3.60–4.10)	3.70 (3.50–4.00)	3.75 (3.40–4.00)	0.016
INR	1.00 (0.95–1.05)	0.98 (0.94–1.03)	1.02 (0.96–1.06)	1.01 (0.95–1.06)	1.00 (0.95–1.07)	0.050
aPTT, s	31.70 (28.50–34.85)	33.00 (30.40–36.70)	31.75 (28.80–34.40)	32.15 (29.30–35.80)	29.70 (25.80–32.10)	< 0.001
Comorbidities						
MI, *n* (%)	13.00 (3.07%)	3.00 (2.83%)	3.00 (2.83%)	2.00 (1.89%)	5.00 (4.72%)	0.680
HF, *n* (%)	9.00 (2.12%)	1.00 (0.94%)	1.00 (0.94%)	2.00 (1.89%)	5.00 (4.72%)	0.181
Hypertension, *n* (%)	232.00 (54.72%)	50.00 (47.17%)	49.00 (46.23%)	65.00 (61.32%)	68.00 (64.15%)	0.011
LD, *n* (%)	55.00 (12.97%)	8.00 (7.55%)	15.00 (14.15%)	15.00 (14.15%)	17.00 (16.04%)	0.272
DM, *n* (%)	59.00 (13.92%)	3.00 (2.83%)	19.00 (17.92%)	11.00 (10.38%)	26.00 (24.53%)	< 0.001
RD, *n* (%)	32.00 (7.55%)	9.00 (8.49%)	6.00 (5.66%)	7.00 (6.60%)	10.00 (9.43%)	0.717
Sepsis, *n* (%)	5.00 (1.18%)	1.00 (0.94%)	1.00 (0.94%)	2.00 (1.89%)	1.00 (0.94%)	0.895
Treatment						
Statin, *n* (%)	85.00 (20.05%)	33.00 (31.13%)	21.00 (19.81%)	20.00 (18.87%)	11.00 (10.38%)	0.002
Vasopressor, *n* (%)	40.00 (9.43%)	3.00 (2.83%)	10.00 (9.43%)	7.00 (6.60%)	20.00 (18.87%)	< 0.001
Insulin, *n* (%)	66.00 (15.57%)	6.00 (5.66%)	17.00 (16.04%)	14.00 (13.21%)	29.00 (27.36%)	< 0.001
MV, *n* (%)	304.00 (71.70%)	73.00 (68.87%)	79.00 (74.53%)	78.00 (73.58%)	74.00 (69.81%)	0.751
Outcomes						
28-day mortality, *n* (%)	38.00 (8.96%)	3.00 (2.83%)	5.00 (4.72%)	7.00 (6.60%)	23.00 (21.70%)	< 0.001
Hospital mortality, *n* (%)	15.00 (3.54%)	1.00 (0.94%)	2.00 (1.89%)	0.00 (0.00%)	12.00 (11.32%)	< 0.001
LOS hospital, days	10.00 (7.00–16.00)	8.00 (5.00–14.00)	11.00 (7.00–16.00)	11.00 (8.00–16.00)	12.00 (5.00–20.00)	0.005

LGI, leuko-glycemic index; GCS, Glasgow Coma Scale; SBP, systolic blood pressure; DBP, diastolic blood pressure; MBP, mean blood pressure; RR, respiratory rate; WBC, white blood cell; BUN, blood urea nitrogen; INR, international normalized ratio; aPTT, activated partial thromboplastin time; LOS, length of stay; MI, myocardial infarction; HF, heart failure; LD, liver disease; DM, diabetes mellitus; RD, renal disease; MV, mechanical ventilation.

In the MIMIC-IV cohort (Table 1), higher LGI quartiles were associated with a progressively more adverse clinical profile. Patients in Q3–Q4 had higher heart rates and respiratory rates and slightly lower systolic, diastolic, and mean blood pressures compared with those in Q1. Laboratory parameters showed a stepwise increase in WBC, blood glucose, blood urea nitrogen, serum creatinine, anion gap, and platelet count across LGI categories, accompanied by lower bicarbonate levels. The prevalence of major comorbidities, including myocardial infarction, heart failure, renal disease, and sepsis, also increased with rising LGI. Consistently, the use of vasopressors, insulin, and mechanical ventilation was more frequent in the higher LGI groups, and patients in Q4 experienced the highest in-hospital, 28-day, and 365-day mortality rates, as well as the longest hospital length of stay.

In the institutional cohort (Table 2), the overall median LGI at admission was 1.13 (IQR 0.80–1.72). Age and sex distribution were broadly similar across LGI quartiles. However, higher LGI was associated with more pronounced physiological and biochemical derangements. Patients in Q3–Q4 had higher WBC counts, blood glucose levels, and anion gap values and lower bicarbonate concentrations compared with those in Q1, and heart rate as well as systolic and mean blood pressure tended to be higher in the upper LGI quartiles. The prevalence of hypertension and diabetes mellitus tended to be higher in the upper LGI quartiles, with the highest proportions observed in Q4. The use of vasopressors and insulin therapy was also more frequent in patients with higher LGI, particularly in Q4. Consistent with these patterns, 28-day mortality, in-hospital mortality, and hospital length of stay were highest in Q4.

### 3.2 Survival analysis

**Figure 2:**
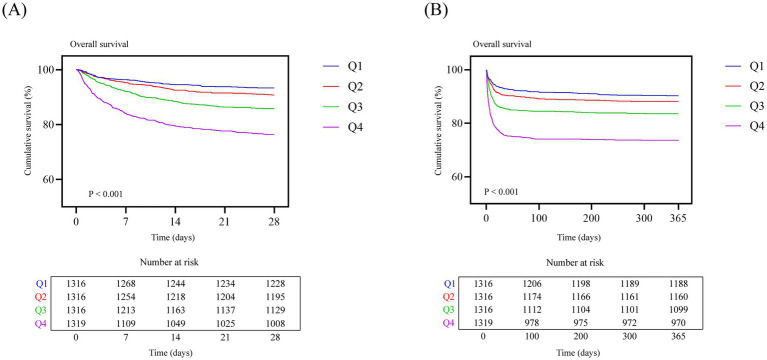
Kaplan–Meier survival curves for 28-day **(A)** and 365-day **(B)** all-cause mortality according to LGI quartiles in the MIMIC-IV cohort. The table below each panel shows the number of patients at risk at different time points. LGI, leuko-glycemic index.

Kaplan–Meier survival curves based on LGI quartiles in the MIMIC-IV cohort are presented in Figure 2. The incidence of the primary outcome differed significantly across LGI groups. Over the first 28 days of follow-up, patients with higher LGI values showed progressively lower survival probabilities, with 28-day mortality increasing stepwise from Q1 to Q4 (log-rank *p* < 0.001; Figure 2A). A similar pattern was observed for long-term outcomes. During 365 days of follow-up, the Kaplan–Meier curves continued to diverge across LGI quartiles, and patients in the highest LGI group (Q4) experienced the poorest survival, whereas those in the lowest LGI group (Q1) had the most favorable prognosis (log-rank *p* < 0.001; Figure 2B). In the institutional cohort, Kaplan–Meier curves for 28-day mortality across LGI quartiles (Supplementary Figure 1) showed a similar pattern, with progressively lower survival in higher LGI groups.

### 3.3 Association between LGI and outcome events

**Figure 3:**
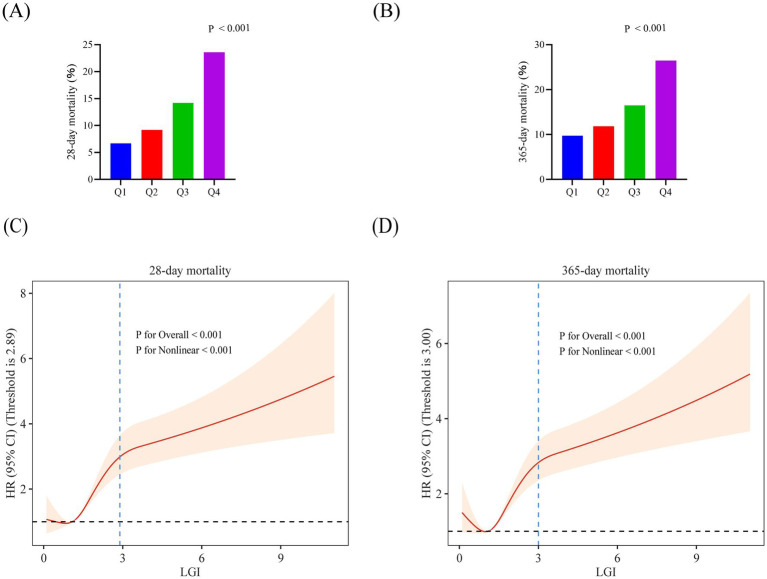
Association between LGI and all-cause mortality in critically ill patients with acute stroke in the MIMIC-IV cohort. **(A)** Comparison of 28-day all-cause mortality across LGI quartiles. **(B)** Comparison of 365-day all-cause mortality across LGI quartiles. **(C,D)** Restricted cubic spline (RCS) curves showing the adjusted HR for all-cause mortality across the continuous LGI range. The vertical dashed lines indicate the inflection points identified by the piecewise Cox regression models with a single change point (Table 4), overlaid on the RCS curves for visual reference. Solid lines represent the fully adjusted HR, shaded areas represent 95% CI, and the horizontal dashed line indicates the reference HR of 1.0. **(C)** RCS for 28-day mortality. **(D)** RCS for 365-day mortality. LGI, leuko-glycemic index; HR, hazard ratio; CI, confidence interval.

The proportional hazards assumption was assessed using Schoenfeld residual plots. For the primary 28-day mortality Cox model (Model 3) with LGI entered as a continuous term, the Schoenfeld residual diagnostics suggested no meaningful violation of the proportional hazards assumption in either cohort (Supplementary Figure 2); the Schoenfeld residual-based test for the LGI term was not significant in the MIMIC-IV cohort (*p* = 0.111) or the institutional cohort (*p* = 0.794). In the MIMIC-IV cohort, higher LGI levels were consistently associated with increased risks of both 28-day and 365-day all-cause mortality, as summarized in Table 3 and depicted in Figures 3A,B. Crude 28-day mortality increased from 6.69% in Q1 to 23.58% in Q4, and 365-day mortality from 9.73 to 26.46%, with the highest event rates consistently observed in the Q4 group. When LGI was modeled as a continuous variable, each 1-unit increase in LGI was associated with a higher hazard of death in the fully adjusted Cox model (Model 3) for both 28-day mortality (HR 1.179, 95% CI 1.150–1.209, *p* < 0.001) and 365-day mortality (HR 1.169, 95% CI 1.142–1.197, *p* < 0.001). Compared with patients in the lowest LGI quartile (Q1), those in higher quartiles had progressively elevated risks of death. In Model 3, the HRs for 28-day mortality were 1.244 (95% CI 0.945–1.638) for Q2, 1.834 (95% CI 1.420–2.367) for Q3, and 3.259 (95% CI 2.568–4.138) for Q4 (*p* for trend < 0.001). A similar graded pattern was observed for 365-day mortality, with HRs of 1.143 (95% CI 0.904–1.445) for Q2, 1.561 (95% CI 1.252–1.946) for Q3, and 2.703 (95% CI 2.203–3.317) for Q4 (*p* for trend < 0.001). In a complete-case sensitivity analysis of the MIMIC-IV cohort (*n* = 3,764; 71.46% of the original cohort), the association between LGI and mortality remained consistent (Supplementary Table 2). In the fully adjusted model (Model 3), the HRs for 28-day mortality across LGI quartiles were 1.288 (95% CI 0.933–1.777) for Q2, 1.894 (95% CI 1.406–2.551) for Q3, and 3.020 (95% CI 2.277–4.006) for Q4 (*p* for trend < 0.001). Similarly, the HRs for 365-day mortality were 1.147 (95% CI 0.878–1.500) for Q2, 1.502 (95% CI 1.166–1.935) for Q3, and 2.414 (95% CI 1.904–3.061) for Q4 (*p* for trend < 0.001).

**Table 3:** Cox proportional hazards models for all-cause mortality.

Characteristic	Continuous	Quartiles of LGI				*p* for trend
		Q1	Q2	Q3	Q4	
28-day mortality in the MIMIC-IV cohort						
Model 1	1.183 (1.157–1.210)*	Reference	1.388 (1.055–1.827)*	2.206 (1.712–2.842)*	3.930 (3.101–4.979)*	< 0.001
Model 2	1.179 (1.153–1.205)*	Reference	1.396 (1.060–1.837)*	2.255 (1.750–2.906)*	4.012 (3.166–5.084)*	< 0.001
Model 3	1.179 (1.150–1.209)*	Reference	1.244 (0.945–1.638)	1.834 (1.420–2.367)*	3.259 (2.568–4.138)*	< 0.001
365-day mortality in the MIMIC-IV cohort						
Model 1	1.175 (1.149–1.201)*	Reference	1.234 (0.977–1.559)	1.773 (1.425–2.206)*	3.083 (2.517–3.775)*	< 0.001
Model 2	1.171 (1.145–1.196)*	Reference	1.240 (0.982–1.567)	1.810 (1.455–2.253)*	3.142 (2.566–3.848)*	< 0.001
Model 3	1.169 (1.142–1.197)*	Reference	1.143 (0.904–1.445)	1.561 (1.252–1.946)*	2.703 (2.203–3.317)*	< 0.001
28-day mortality in the institutional cohort						
Model 1	1.605 (1.363–1.889)*	Reference	1.678 (0.401–7.023)	2.327 (0.602–9.000)	8.548 (2.566–28.472)*	< 0.001
Model 2	1.610 (1.368–1.896)*	Reference	1.531 (0.364–6.435)	2.206 (0.570–8.538)	8.566 (2.567–28.584)*	< 0.001
Model 3	1.351 (1.090–1.675)*	Reference	1.542 (0.367–6.477)	1.646 (0.419–6.466)	4.374 (1.270–15.063)*	0.004

Model 1: unadjusted. Model 2: adjusted for age and sex. Model 3: adjusted for age, sex, Glasgow Coma Scale, and mechanical ventilation. *p < 0.05. For the institutional cohort, quartile-based estimates should be interpreted with caution because of the relatively small sample size and limited number of events.

In the institutional cohort, the direction of association between LGI and 28-day mortality was concordant with that observed in the MIMIC-IV cohort (Table 3). In the fully adjusted model (Model 3), LGI as a continuous variable remained significantly associated with 28-day mortality (HR 1.351, 95% CI 1.090–1.675, *p* < 0.05). Using Q1 as the reference, patients in Q4 had a markedly higher risk of 28-day death (HR 4.374, 95% CI 1.270–15.063), while HRs for Q2 and Q3 were 1.542 (95% CI 0.367–6.477) and 1.646 (95% CI 0.419–6.466), respectively (*p* for trend = 0.004). Although the CIs were wider than in the MIMIC-IV cohort—reflecting the smaller sample size and fewer events—the overall pattern supported a positive, dose–response relationship between LGI and short-term mortality in the external cohort. Because long-term (365-day) follow-up data were not available for the institutional cohort, only the primary outcome of 28-day all-cause mortality was evaluated in this cohort. In the institutional cohort, quartile-based estimates warrant cautious interpretation given the relatively small sample size and limited events.

To further describe the association between LGI and mortality, RCS analyses were performed in the MIMIC-IV cohort (Figures 3C,D). These analyses revealed a positive but nonlinear association between LGI and mortality, with risk rising steeply from low to moderate LGI levels and then increasing more gradually at higher levels. In the piecewise Cox proportional hazards models with a single change point, the estimated inflection points were 2.89 for 28-day mortality and 3.00 for 365-day mortality (Table 4). Below these points, LGI showed a strong association with mortality (28-day mortality: HR 1.95, 95% CI 1.77–2.15; 365-day mortality: HR 1.72, 95% CI 1.58–1.88; both *p* < 0.001), whereas above these inflection points the slope of the association was markedly attenuated (28-day mortality: HR 1.05, 95% CI 1.00–1.11, *p* = 0.045; 365-day mortality: HR 1.06, 95% CI 1.02–1.11, *p* = 0.009). The log-likelihood ratio tests indicated that the two-segment models provided a significantly better fit than a single linear term for LGI (both *p* < 0.001).

**Table 4:** Threshold effect analysis in the MIMIC-IV cohort.

Model/Segment	28-day mortality	365-day mortality
Cox model with LGI as a linear term	1.18 (1.15–1.21) *p* < 0.001	1.17 (1.15–1.20) *p* < 0.001
Inflection point (K) in piecewise Cox model	2.89	3.00
< K	1.95 (1.77–2.15) *p* < 0.001	1.72 (1.58–1.88) *p* < 0.001
> K	1.05 (1.00–1.11) *p* = 0.045	1.06 (1.02–1.11) *p* = 0.009
log-likelihood ratio test	*p* < 0.001	*p* < 0.001

### 3.4 Subgroup analysis

**Figure 4:**
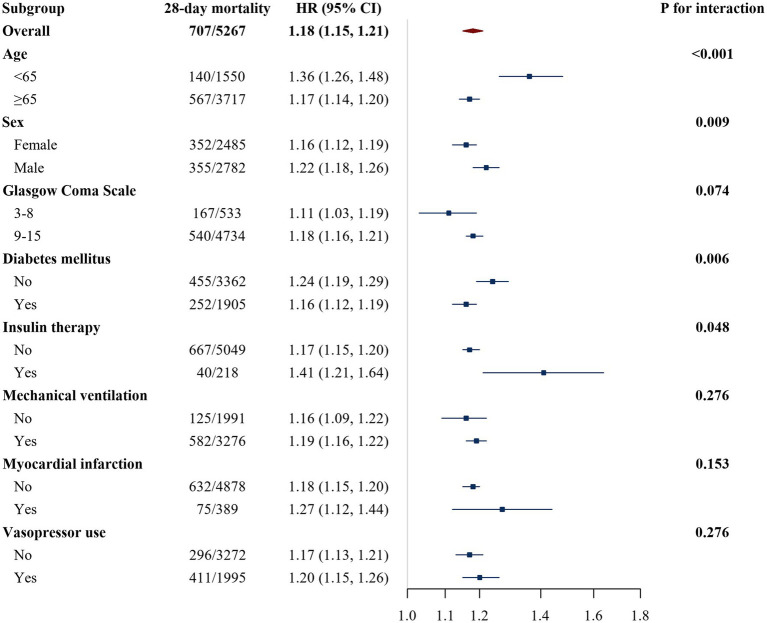
Subgroup analysis of the association between LGI and 28-day all-cause mortality in the MIMIC-IV cohort. The HR represents the risk per 1-unit increase in the leuko-glycemic index (LGI) within each subgroup. Estimates are HRs and 95% CIs derived from Cox proportional hazards regression models (Model 2, as defined in Methods). In each subgroup analysis, the subgroup variable was not additionally adjusted for. The *p* value for interaction tests whether the association between LGI and 28-day mortality differs across categories of age (<65 vs. ≥65 years), sex, GCS score (≤8 vs. >8), myocardial infarction, diabetes mellitus, vasopressor use, insulin therapy, or mechanical ventilation. LGI, leuko-glycemic index; HR, hazard ratio; CI, confidence interval.

**Figure 5:**
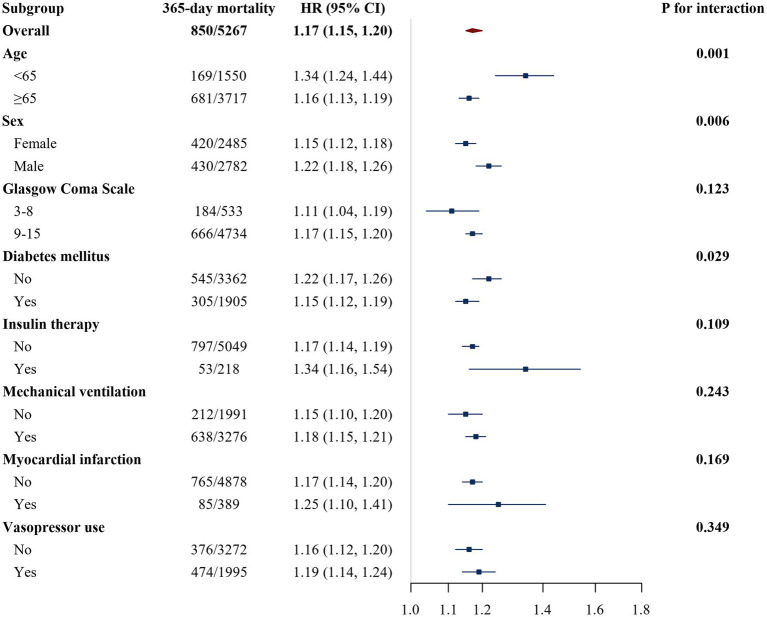
Subgroup analysis of the association between LGI and 365-day all-cause mortality in the MIMIC-IV cohort. The HR represents the risk per 1-unit increase in the leuko-glycemic index (LGI) within each subgroup. Estimates are HRs and 95% CIs derived from Cox proportional hazards regression models (model 2, as defined in Methods). In each subgroup analysis, the subgroup variable was not additionally adjusted for. The *p* value for interaction tests whether the association between LGI and 365-day mortality differs across categories of age (<65 vs. ≥65 years), sex, GCS score (≤8 vs. >8), myocardial infarction, diabetes mellitus, vasopressor use, insulin therapy, or mechanical ventilation. LGI, leuko-glycemic index; HR, hazard ratio; CI, confidence interval.

To evaluate the robustness of the association between LGI and mortality and to explore potential effect modification, subgroup analyses with interaction tests were performed in the MIMIC-IV cohort, stratified by age, sex, GCS score, history of myocardial infarction, diabetes mellitus, vasopressor use, insulin therapy, and mechanical ventilation status (Figures 4, 5). For 28-day mortality, higher LGI was consistently associated with an increased risk of death across all strata, with HR remaining above 1.0 in each subgroup. The strength of this association varied, with statistically significant effect modification observed for age (*p* for interaction < 0.001), sex (*p* for interaction = 0.009), diabetes mellitus (*p* for interaction = 0.006), and insulin therapy (*p* for interaction = 0.048). In general, the association between LGI and 28-day mortality appeared stronger among younger patients (<65 years), men, individuals without diabetes, and those receiving insulin therapy. No significant effect modification was detected for GCS score, myocardial infarction, vasopressor use, or mechanical ventilation status with respect to 28-day mortality (all *p* for interaction > 0.05).

For 365-day all-cause mortality, elevated LGI was likewise associated with higher risk in all examined subgroups. Significant effect modification was again noted for age (*p* for interaction = 0.001), sex (*p* for interaction = 0.006), and diabetes mellitus (*p* for interaction = 0.029), with a generally stronger association in younger patients, men, and those without diabetes, whereas no statistically significant effect modification was found for GCS score, myocardial infarction, vasopressor use, mechanical ventilation, or insulin therapy (all *p* for interaction > 0.05).

Because the number of patients with very high LGI values above the estimated change points from the threshold-effect analysis (approximately 2.89 for 28-day mortality and 3.00 for 365-day mortality) was limited, additional sensitivity analyses were conducted by restricting the MIMIC-IV cohort to patients with LGI values below these change points (Supplementary Figures 3, 4). Within this main exposure range, the overall positive association between LGI and both 28-day and 365-day mortality remained evident, and in many subgroups the HRs continued to suggest higher risk with increasing LGI, although in some strata the estimates were closer to unity or even slightly below 1.0. For 28-day mortality, the effect modification by age, sex, diabetes mellitus, and insulin therapy that was statistically significant in the primary analyses was no longer statistically significant in the restricted analyses, whereas interactions with GCS score and vasopressor use reached nominal significance. For 365-day mortality, after restriction to LGI values below the change point, interaction terms for age and sex were no longer statistically significant, whereas the interaction with diabetes mellitus remained significant, with the direction of effect differing from the primary analysis, and interaction *p* values for insulin therapy and vasopressor use were also < 0.05. These shifts in *p* for interaction likely reflect the reduced number of events and narrower exposure range in the restricted cohort and therefore should be interpreted cautiously. Overall, despite some inconsistencies in interaction patterns, the subgroup and sensitivity analyses support LGI as a robust prognostic marker in critically ill stroke patients, while suggesting potential heterogeneity of effects across demographic and metabolic strata. All interaction analyses were considered exploratory, and no formal adjustment for multiple comparisons was performed.

## 4 Discussion

In this study, we found that LGI was significantly associated with mortality in critically ill stroke patients across two independent cohorts. Our findings indicate that elevated LGI is significantly associated with higher 28-day and 365-day all-cause mortality after multivariable adjustment, supporting its value as a practical marker reflecting combined inflammatory burden and stress hyperglycemia. In descriptive analyses, in-hospital mortality was also highest in the top LGI quartile in both cohorts. Notably, the LGI–mortality association was not strictly linear; instead, it followed a nonlinear pattern, with risk rising steeply from low to moderate LGI values and then increasing more gradually at higher levels. It should be noted that the inflection points identified in the threshold analyses represent statistical approximations rather than exact biological cutoffs, and are intended to facilitate clinical interpretation of the nonlinear patterns observed in the spline models. In our data, these inflection points were located around LGI values of 2.9–3.0, roughly corresponding to the higher end of the LGI distribution, suggesting that most of the prognostic information is concentrated within a moderate range of LGI values before risk begins to plateau. At very low LGI values, the confidence intervals were wide and encompassed unity, suggesting limited precision of the risk estimates in this extreme range. Findings from the external institutional cohort were directionally consistent, with higher LGI associated with higher 28-day and in-hospital mortality and a significant effect when modeled continuously; however, only the top LGI quartile reached statistical significance and confidence intervals were wide, reflecting the small sample size and limited events, so estimates from this cohort should be interpreted with caution. Collectively, these data suggest that LGI may be a useful tool for early risk stratification in critically ill stroke patients, a setting in which accessible and reliable prognostic indicators remain limited.

LGI has recently emerged as a composite biomarker that integrates information on systemic inflammation and glycemic disturbance and has been linked to prognosis in conditions such as coronary artery disease, COVID-19, and trauma (8–10, 22). Conceptually, LGI captures two central components of the acute stress response—leukocytosis and hyperglycemia—which often occur simultaneously and interact biologically. Observational studies have shown that both elevated WBC and stress hyperglycemia are associated with worse outcomes after stroke, including larger infarct volumes, higher rates of hemorrhagic transformation, poorer functional recovery, and greater short- and long-term mortality (23–26). From a mechanistic perspective, leukocytosis reflects activation of innate immunity and cytokine cascades; infiltrating neutrophils and monocytes can promote microvascular plugging, endothelial dysfunction, and release of reactive oxygen species and proteolytic enzymes, thereby intensifying secondary brain injury and peri-infarct inflammation (27). Meanwhile, hyperglycemia amplifies oxidative stress, enhances leukocyte adhesion and migration, and exacerbates blood–brain barrier disruption and cerebral edema (28–32). Importantly, these two processes may reinforce each other: inflammation induces insulin resistance and neuroendocrine stress activation, whereas hyperglycemia amplifies inflammatory signaling and leukocyte adhesion, forming a vicious cycle. Thus, the association between LGI and mortality observed in our study is biologically plausible and supports the idea that LGI reflects the synergistic inflammatory–metabolic burden after acute stroke, providing an integrated marker of systemic stress that is particularly relevant in critically ill patients.

Beyond reflecting two parallel stress pathways, an elevated LGI may also indicate a pathophysiological milieu in which inflammation and dysglycemia mutually reinforce each other after acute stroke. Systemic inflammatory activation can trigger neuroendocrine responses and cytokine-mediated insulin resistance, promoting stress hyperglycemia (33–36), whereas hyperglycemia further potentiates inflammatory signaling (37, 38), creating a self-amplifying cycle. This interaction may accelerate ischemic and hemorrhagic injury through several mechanisms: heightened pro-thrombotic tendency and platelet activation, impaired microcirculatory perfusion, and greater vulnerability of the blood–brain barrier to oxidative and inflammatory damage (39–42). In addition, leukocyte-endothelial interactions and glucose-driven metabolic toxicity may worsen cerebral edema, increase intracranial pressure, and predispose to secondary complications such as infection or multi-organ dysfunction in the ICU setting (43–45). Therefore, LGI likely serves not only as a composite snapshot of acute physiological stress but also as a surrogate of the intertwined inflammatory-metabolic cascade that contributes to early neurological deterioration and long-term mortality. These mechanistic considerations provide a rationale for the heterogeneity of the LGI–mortality association observed across clinical subgroups.

Subgroup analyses suggested that the prognostic effect of LGI on mortality was not entirely uniform across patient strata (Figures 4, 5). For both 28-day and 365-day mortality, the positive association between LGI and risk of death was present in all examined subgroups, but tended to be stronger in younger patients (<65 years), in men, and in patients without diabetes mellitus, with statistically significant interactions for age, sex, and diabetes mellitus. In addition, for 28-day mortality the association appeared more pronounced among patients receiving insulin therapy. No meaningful effect modification was observed for GCS score, history of myocardial infarction, vasopressor use, or mechanical ventilation status. Taken together, these findings reinforce LGI as a broadly useful marker while suggesting that its risk signal may be especially strong in younger, male, nondiabetic, and insulin-treated patients; however, these apparent differences should be interpreted cautiously because several subgroups, particularly those defined by specific treatments, contained relatively few events, which limits the precision of the interaction estimates. From a clinical standpoint, our results highlight LGI as a potentially useful and easily accessible tool for identifying high-risk patients among those admitted to the ICU with stroke. Because LGI is calculated from WBC and blood glucose measurements that are universally available in emergency and critical care settings, it could be readily incorporated into existing assessment protocols without additional cost or testing burden. Patients with markedly elevated LGI might benefit from closer monitoring, more aggressive management of hyperglycemia and systemic inflammation, and heightened vigilance for secondary complications. At the same time, LGI should not be regarded as a stand-alone diagnostic criterion but rather as a complementary marker that adds incremental prognostic information to clinical judgment, imaging findings, and validated severity scores. Future work is needed to determine whether interventions that modulate components of LGI—such as optimized glycemic control or targeted anti-inflammatory strategies—can translate into improved outcomes in this high-risk population. These patterns were generally preserved in sensitivity analyses restricted to patients with LGI values below the estimated change points (Supplementary Figures 3, 4). Within this main exposure range, the overall pattern of a positive association between LGI and both 28-day and 365-day mortality was preserved, with hazard ratios generally above 1.0 across most examined subgroups, whereas several interaction terms that were statistically significant in the full cohort lost significance and a few new nominal interactions emerged. This behavior suggests that at least part of the apparent effect modification by age, sex, diabetes mellitus, insulin therapy, and vasopressor therapy may be driven by patients with extreme LGI values in the full cohort and by the reduced number of outcome events within certain strata in the restricted analyses, both of which make the interaction estimates less stable. Accordingly, all subgroup findings should be regarded as exploratory and hypothesis-generating rather than definitive.

Several limitations of this study should be acknowledged. First, the retrospective observational design inherently limits causal inference, and residual confounding cannot be excluded despite extensive multivariable adjustment and subgroup analyses. Important variables such as detailed stroke subtype classification, baseline neurological severity scores, hematoma volume, and information on pre-hospital management were not available or not systematically recorded in the databases used, and as with all database studies, misclassification of diagnoses and comorbidities based on coding or documentation errors may have occurred. Second, LGI was evaluated only at admission, without examining dynamic changes or their prognostic significance. Future studies should explore alternative summaries (e.g., peak or mean values) to further assess robustness. Third, given the limited number of death events in the institutional cohort, we intentionally applied a parsimonious multivariable model; nevertheless, reduced statistical power and potential overfitting cannot be completely excluded. Fourth, both cohorts were derived from tertiary care centers, and our findings may not be fully generalizable to less severely ill stroke populations or other healthcare systems. Fifth, the subgroup analyses assumed a linear relationship between LGI and mortality within strata, whereas the overall relationship was nonlinear, which may affect the interpretation of interaction terms.

## 5 Conclusion

LGI was significantly associated with mortality in critically ill stroke patients across two independent cohorts. As a simple and readily available biomarker, LGI may aid early risk stratification and identify patients at higher risk who warrant closer clinical attention. Prospective multicenter studies should validate its clinical utility.

## Data Availability

The raw data supporting the conclusions of this article will be made available by the authors, without undue reservation.
