# Supplementary material for: Association of leuko-glycemic index with mortality in critically ill stroke patients: analysis from the MIMIC-IV database and an institutional cohort
Source: Front Med (Lausanne). 2026 Feb 11;13:1755389. doi: 10.3389/fmed.2026.1755389 (PMC12932427; doi:10.3389/fmed.2026.1755389)
Supplement: Supplementary file 1 [file Data_Sheet_1.pdf]

Supplementary Materials

1 Supplementary Tables

Supplementary Table 1 Summary of missing data for variables in the MIMIC-IV cohort (N = 5,267)

Variable	Missing, n (%)	Variable	Missing, n (%)
LGI	0 (0.00%)	Sodium	1 (0.02%)
Age	0 (0.00%)	Potassium	2 (0.04%)
Male	0 (0.00%)	INR	643 (12.21%)
GCS	22 (0.42%)	aPTT	673 (12.78%)
Heart rate	9 (0.17%)	MI	0 (0.00%)
SBP	475 (9.02%)	HF	0 (0.00%)
DBP	475 (9.02%)	Hypertension	0 (0.00%)
MBP	476 (9.04%)	LD	0 (0.00%)
RR	18 (0.34%)	DM	0 (0.00%)
Temperature	318 (6.04%)	RD	0 (0.00%)
Hemoglobin	1 (0.02%)	Sepsis	0 (0.00%)
Platelet count	1 (0.02%)	Statin	0 (0.00%)
WBC	0 (0.00%)	Vasopressor	0 (0.00%)
Anion gap	4 (0.08%)	Insulin	0 (0.00%)
Bicarbonate	0 (0.00%)	MV	0 (0.00%)
BUN	5 (0.09%)	28-day mortality	0 (0.00%)

Serum creatinine	1 (0.02%)	365-day mortality	0 (0.00%)
Calcium	309 (5.87%)	Hospital mortality	0 (0.00%)
Chloride	2 (0.04%)	LOS hospital	0 (0.00%)
Glucose	0 (0.00%)	Height (excluded due to missingness)	2,857 (54.24%)

Abbreviations: LGI, leuko-glycemic index; GCS, Glasgow Coma Scale; SBP, systolic blood pressure; DBP, diastolic blood pressure; MBP, mean blood pressure; RR, respiratory rate; WBC, white blood cell; BUN, blood urea nitrogen; INR, international normalized ratio; aPTT, activated partial thromboplastin time; LOS, length of stay; MI, myocardial infarction; HF, heart failure; LD, liver disease; DM, diabetes mellitus; RD, renal disease; MV, mechanical ventilation.

Supplementary Table 2 Cox proportional hazards models for all-cause mortality (Complete-case analysis; N = 3,764)

Characteristic	Continuous	Quartiles of LGI				P for trend
		Q1	Q2	Q3	Q4	
**28-day mortality in the MIMIC-IV cohort**						
Model 1	1.169 (1.139–1.200)*	Reference	1.456 (1.056–2.007)*	2.449 (1.824–3.289)*	3.988 (3.016–5.273)*	< 0.001
Model 2	1.164 (1.134–1.195)*	Reference	1.438 (1.043–1.983)*	2.455 (1.828–3.297)*	3.978 (3.008–5.259)*	< 0.001
Model 3	1.149 (1.114–1.185)*	Reference	1.288 (0.933–1.777)	1.894 (1.406–2.551)*	3.020 (2.277–4.006)*	< 0.001
**365-day mortality in the MIMIC-IV cohort**						
Model 1	1.161 (1.132–1.191)*	Reference	1.262 (0.967–1.649)	1.813 (1.412–2.328)*	2.978 (2.357–3.763)*	< 0.001
Model 2	1.156 (1.127–1.186)*	Reference	1.250 (0.957–1.633)	1.819 (1.416–2.336)*	2.972 (2.352–3.755)*	< 0.001
Model 3	1.142 (1.109–1.175)*	Reference	1.147 (0.878–1.500)	1.502 (1.166–1.935)*	2.414 (1.904–3.061)*	< 0.001

Model 1: unadjusted. Model 2: adjusted for age and sex. Model 3: adjusted for age, sex, Glasgow Coma Scale, and mechanical ventilation. *P < 0.05. This sensitivity analysis was conducted as a complete-case analysis, excluding participants with missing covariates included in the multivariable models. For comparability across models, Models 1 – 3 were all fitted in the same complete-case subset defined by complete data on all covariates included in Model 3 (N = 3,764).

2 Supplementary Figures

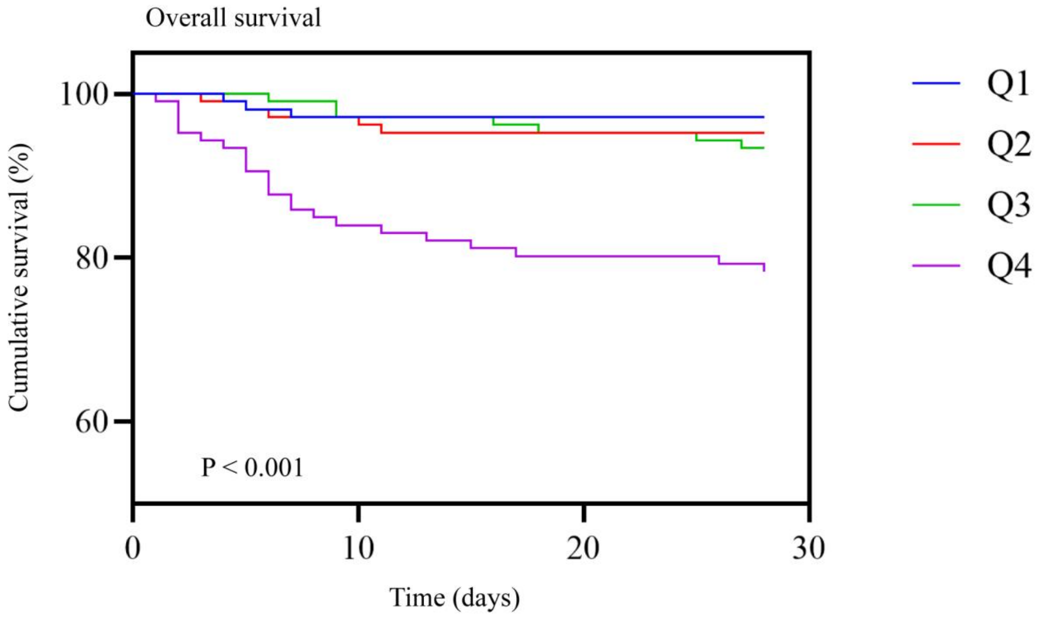

Number at risk

Q1	106	104	103	103	103
Q2	106	103	101	101	101
Q3	106	105	103	101	99
Q4	106	93	87	85	84
	0	7	14	21	28

Time (days)

Supplementary Figure 1 Kaplan–Meier survival curves for 28-day all-cause mortality according to LGI quartiles in the institutional cohort. The table below the curve shows the number of patients at risk at different time points. LGI, leuko-glycemic index.

(A) (B)

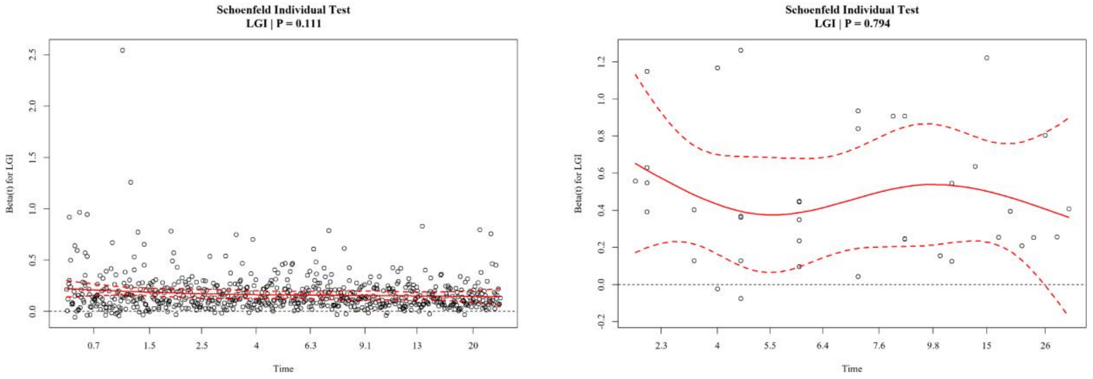

Supplementary Figure 2 Schoenfeld residual plots for LGI in the MIMIC-IV cohort (A) and the institutional cohort (B). Plots correspond to the primary 28-day mortality Cox model (Model 3) with LGI entered as a continuous term. The P values shown in each panel correspond to the Schoenfeld residual-based test for the LGI term (MIMIC-IV: P = 0.111; institutional cohort: P = 0.794), indicating no strong evidence against the proportional hazards assumption. The solid red lines represent smoothed trends of the scaled Schoenfeld residuals over time, and the dashed red lines indicate 95% confidence bands. LGI, leuko-glycemic index.

Subgroup	28-day mortality	HR (95% CI)	P for interaction
**Overall**	**538/4679**	**1.96 (1.73, 2.23)**	
**Age**			**0.562**
<65	104/1395	2.13 (1.60, 2.84)	
≥65	434/3284	1.94 (1.68, 2.24)	
**Sex**			**0.962**
Female	267/2193	1.96 (1.64, 2.35)	
Male	271/2486	1.96 (1.63, 2.36)	
**Glasgow Coma Scale**			**0.019**
3-8	127/447	1.40 (1.06, 1.84)	
9-15	411/4232	2.03 (1.75, 2.35)	
**Diabetes mellitus**			**0.063**
No	356/3009	1.80 (1.54, 2.11)	
Yes	182/1670	2.33 (1.86, 2.91)	
**Insulin therapy**			**0.073**
No	522/4537	2.01 (1.76, 2.29)	
Yes	16/142	1.00 (0.46, 2.18)	
**Mechanical ventilation**			**0.908**
No	102/1819	1.81 (1.35, 2.43)	
Yes	436/2860	1.83 (1.58, 2.12)	
**Myocardial infarction**			**0.333**
No	484/4367	1.90 (1.66, 2.18)	
Yes	54/312	2.49 (1.60, 3.87)	
**Vasopressor use**			**0.001**
No	230/2988	2.29 (1.89, 2.78)	
Yes	308/1691	1.44 (1.20, 1.73)	

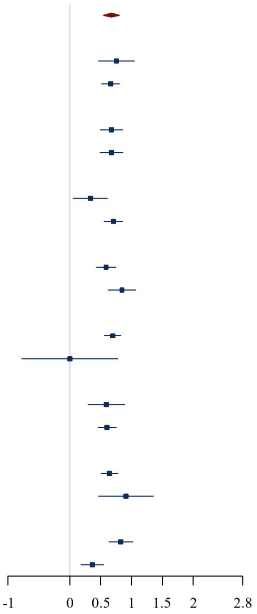

Supplementary Figure 3 Sensitivity analysis of the association between LGI and 28-day all-cause mortality in the MIMIC-IV cohort, restricted to patients with LGI values below the threshold identified in the two-piecewise Cox model (K = 2.89). The HR represents the risk per 1-unit increase in the leuko-glycemic index (LGI) within each subgroup. Estimates are HRs and 95% CIs derived from Cox proportional hazards regression models (Model 2, as defined in Methods). In each subgroup analysis, the subgroup variable was not additionally adjusted for. The P value for interaction tests whether the association between LGI and 28-day mortality differs across categories of age (<65 vs. ≥65 years), sex, GCS score (≤8 vs. >8), myocardial infarction, diabetes mellitus, vasopressor use, insulin therapy, or mechanical ventilation. LGI, leuko-glycemic index; HR, hazard ratio; CI, confidence interval.

Subgroup	365-day mortality	HR (95% CI)	P for interaction
**Overall**	**677/4725**	**1.73 (1.55, 1.94)**	
**Age**			**0.730**
<65	135/1411	1.83 (1.43, 2.34)	
≥65	542/3314	1.73 (1.52, 1.96)	
**Sex**			**0.672**
Female	332/2210	1.68 (1.44, 1.98)	
Male	345/2515	1.78 (1.52, 2.08)	
**Glasgow Coma Scale**			**0.171**
3-8	143/456	1.42 (1.10, 1.82)	
9-15	534/4269	1.73 (1.52, 1.96)	
**Diabetes mellitus**			**0.041**
No	444/3035	1.59 (1.38, 1.83)	
Yes	233/1690	2.02 (1.67, 2.45)	
**Insulin therapy**			**0.046**
No	652/4580	1.77 (1.58, 1.98)	
Yes	25/145	0.97 (0.53, 1.76)	
**Mechanical ventilation**			**0.241**
No	177/1832	1.49 (1.20, 1.86)	
Yes	500/2893	1.71 (1.50, 1.96)	
**Myocardial infarction**			**0.306**
No	615/4411	1.69 (1.50, 1.90)	
Yes	62/314	2.20 (1.46, 3.31)	
**Vasopressor use**			**0.006**
No	310/3011	1.89 (1.61, 2.22)	
Yes	367/1714	1.35 (1.15, 1.59)	

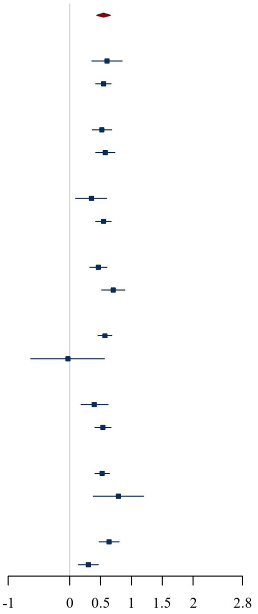

Supplementary Figure 4 Sensitivity analysis of the association between LGI and 365-day all-cause mortality in the MIMIC-IV cohort, restricted to patients with LGI values below the threshold identified in the two-piecewise Cox model (K = 3.00). The HR represents the risk per 1-unit increase in the leuko-glycemic index (LGI) within each subgroup. Estimates are HRs and 95% CIs derived from Cox proportional hazards regression models (Model 2, as defined in Methods). In each subgroup analysis, the subgroup variable was not additionally adjusted for. The P value for interaction tests whether the association between LGI and 365-day mortality differs across categories of age (<65 vs. ≥65 years), sex, GCS score (≤8 vs. >8), myocardial infarction, diabetes mellitus, vasopressor use, insulin therapy, or mechanical ventilation. LGI, leuko-glycemic index; HR, hazard ratio; CI, confidence interval.
